# Depressive symptoms affect short- and long-term speech recognition outcome in cochlear implant users

**DOI:** 10.1007/s00405-020-06096-3

**Published:** 2020-06-05

**Authors:** Katharina Heinze-Köhler, Effi Katharina Lehmann, Ulrich Hoppe

**Affiliations:** CICERO-Cochlear Implant Centrum, ENT-Clinic, Friedrich-Alexander-Universität Erlangen-Nürnberg (FAU), Waldstr. 1, 91054 Erlangen, Germany

**Keywords:** Cochlear implant, Outcome, Speech recognition, Depression

## Abstract

**Purpose:**

To investigate the impact of the amount of depressive symptoms in cochlear implant (CI) recipients on the development of speech recognition after CI-activation up to 2 years.

**Design:**

Retrospective data analysis of a German short form of the Beck Depression Inventory given at initial activation of the implant in relation to monosyllabic word recognition score at conversational level at initial activation and at 3 months, 1 and 2-year follow-up measurements.

**Study sample:**

Thirty-one CI-patients (11 female, 20 male) aged between 41 and 83 (*M* = 64.77, SD = 10.43) who were German native speakers, postlingually deafened, with severe hearing loss in both sides but unilaterally implanted (19 right-sided, 12 left-sided).

**Results:**

The amount of depressive symptoms at initial activation was negatively correlated with the monosyllabic recognition score after 3 months and after 1 year of implant use.

**Conclusion:**

The psychological status in terms of depressive symptoms is an important parameter regarding the rehabilitative outcome of CI-patients. Care staff and CI-users should be sensitized to the link between depressive symptoms and the development of speech recognition with CI.

## Introduction

Hearing loss is a chronic condition with a global high prevalence [1]. Moreover, persons suffering from severe hearing loss are at risk for experiencing social isolation, loneliness, distress and depressive symptoms [e.g., 2, 3]. In a large Norwegian population sample, Tambs [4] reports an increase of 0.1 standard deviations in depression and anxiety symptoms per 10 dB hearing loss.

Profound sensorineural hearing loss can be treated by cochlear implants (CI) when other hearing aids fail to restore functional hearing. Cochlear implantation requires a rehabilitation process, yielding a stable plateau of speech comprehension after 1–2 years [5]. There is evidence that CI-use improves quality of life and depressive symptoms [6–10; but see also 11, who found no changes in a depression subscale].

Besides that, data on the relationship between audiological measures and psychological measures in CI-recipients is controversial. Improvements in subjective hearing in several daily situations and objective recognition of monosyllabic words after cochlear implantation are moderately correlated [12]. In a meta-analysis, McRackan et al. [7] found low overall correlations between self-reported outcome measures including quality of life and psychological measures and word and sentence recognition. However, only one early study using several measures of mental health status was included in the analysis, which reported correlations between monosyllabic word and sentence tests and depressiveness when using mean values of several long-term follow-up measurements between 9 and 54 months [6].

To date, most research has focused on the impact of CI-implantation on psychosocial outcomes and the cross-sectional relationship between psychosocial outcomes and audiological functioning. In return, only few studies tap the influence of psychological status at initial activation on the subsequent objective hearing outcome: Knutson et al. [13] found correlations between pre-implant self-reported participatory compliance and sentence and phoneme tests 18 months post-initial activation. Van Dijk et al. [14] found no predictive impact of personality and handicap attitude questionnaires on audiological outcome. A relationship between hearing-specific quality of life 6 months after implantation and the increase of recognition of disyllabic words after 12 months was found by Mosnier et al. [9]. According to Brüggemann et al. [15], CI-patients with diagnosed psychosomatic disorders before implantation that vanished after implantation improved in monosyllabic perception 6 months after implantation, while patients with persisting psychiatric diagnoses did not. On the other hand, new diagnoses of affective disorders following CI-implantation had no impact on the audiological outcome.

Psychological factors could influence audiological outcome via motivation, compliance, learning and plasticity or habits of all-day use of the CI. In the attempt to establish predictors of CI outcome, only a few factors are confirmed to date, as for instance, duration of hearing loss before implantation, residual hearing, and age at implantation [16, 17]. Regarding psychological factors, more data is needed to assess the effect on audiological outcomes [18]. Depression is one of the most common and most disabling mental disorders worldwide [19]. But besides psychiatric diagnoses on the one hand and quality of life measures on the other, measures of mental health that quantify symptoms in a wide range are of interest. Here, depressive symptoms were quantified with a German short version of the Beck’s Depression Inventory (BDI-V) [20] that allows values between 0 and 100 and indicates a 90% probable depression for values above 35.

The aim of this study was to investigate the development of speech recognition after CI-activation longitudinally up to 2 years, in relation to the amount of depressive symptoms at initial activation.

## Materials and methods

### Sample

Thirty-one CI-patients (11 female, 20 male) aged between 41 and 83 (*M* = 64.77, SD = 10.43) were included in the study (see Table 1).

**Table 1:** Demographic characteristics of participants

Age at initial activation	41-83 (*M* = 64.77, SD = 10.43)
Etiology	14 unknown, 4 sudden hearing loss, 1 Otosclerosis, 1 Menière’s disease, 3 hereditary, 3 noise-induced hearing loss, 2 Meningitis, 3 other infections
Sex	11 female, 20 male
Implantation side	19 right, 12 left

The BDI-V was given to all patients at our center as a routine screening during a stay of 3 days at the initial activation of the CI since midyear 2014. In the study, all patients were included that completed the BDI-V and were at least 1-year post-initial activation until midyear 2016 and that fulfilled the inclusion criteria.

Patients gave written informed consent to statistical analysis of their medical data at the beginning of the rehabilitation, and the retrospective data analysis was approved by a local ethics committee.

All patients were postlingually deafened (for etiology of the hearing loss see Table 1). Note that patients with an unilateral hearing impairment from childhood on who later deafened in both sides as an adult were not excluded. The majority of participants were German native speakers. One participant was bilingual with German as one of two languages with comparable level. Persons with comorbid medical diseases were not excluded as long as the diseases were hypothesized not to interfere with speech recognition. Seven out of 31 patients had no other comorbid disease. Another seven patients exhibited chronic hypertension as the only comorbid disease. In the remaining seventeen patients, there was one bronchial asthma, one hyperuricemia, one sleep apnea, one cirrhosis of the kidney, one skin cancer, two type II diabetes, three hypercholesterolemia, four diseases of the gastrointestinal tract, four orthopedic diseases, five heart diseases, and five with thyroid disorders (multiple diagnoses possible).

To ensure that all measurement timepoints could be clearly assigned to initial activation of one side, and because of the low number of bilaterally implanted adults, only unilaterally implanted patients were included (19 right-sided, 12 left-sided). In turn, only patients with bilateral hearing loss were included, as it was hypothesized that with single sided deafness the relationship between speech comprehension with CI and psychological measures might be different from bilateral hearing loss. A bilateral hearing loss was defined as a preoperative level of maximal 50% recognition of monosyllabic words (Freiburg Monosyllabic Word Test, see below) without hearing aids in the better ear.

For 23 of 31 patients, self-reported durations of severe to profound hearing loss were available. The remaining patients could not give full particulars about their hearing loss in years. Durations varied from 1 to 65 years (*M* = 21.04, SD = 20.25), while the distribution was skewed towards shorter durations (in 10 patients shorter than 10 years).

### Procedure

The BDI-V was completed within 3 days after initial activation during a routine psychological counselling. To patients with a score beyond 35 which corresponds to a high chance of a clinically relevant depression (see below), a professional external intervention was recommended. All audiometric measurements were also part of the routine CI controls. Recognition of monosyllables was first tested between 3 days up to maximally 2 weeks after initial activation of CI.

### Audiological assessment

The recognition of monosyllabic words was operationalized with the Freiburg Monosyllabic Word Test [21]. All measurements were taken monaural with the CI-system. The contralateral ear was deafened with an earplug and headphone-applied noise for adequate masking. The measurements took place in a test room with optimized room acoustics to evaluate a quasi-free field test condition. The sound pressure level was 65 dB SPL. The speakers were positioned in front of the patients at a distance of one meter. The functionality of the CI was checked before the measurements by an audiologist. The Freiburg Monosyllabic Word Test is part of the standard clinical rehabilitation evaluation in our center.

### BDI-V

To measure depressiveness a German short version of the Beck Depression Inventory [20] was used. The original version of the BDI [22] is one of the world's most frequently used instruments for diagnosing depression. Here, 84 items represent a total of 21 typical symptoms of depression, e.g., sadness, irritability, despair and self-reproach. The shortened version contains only 20 items and is, therefore, more economical. Despite this, it places less strain on patients and has the same high quality criteria as the original [23].

The questionnaire was introduced according to the specifications of the developers with the following instruction: "This questionnaire is about your current attitude towards life. For each question, please indicate how often you experience the mentioned mood or point of view". The BDI-V is answered using a six-step frequency scale with numerical step anchoring. The extreme values are also linguistically anchored (0/never—1—2—3—4—5/almost always). The addition of the values across all items results in the total score with a possible value range of 0–100. From a score of 35, there is a 90% probability of clinically relevant depression [24]. Patients with such a value were motivated to take appropriate treatment options.

### Data analyses

All statistical analyses were performed using SPSS Statistics for Windows, Version 21.0 (IBM Corp., Armonk, NY).

All variables were tested for Gaussian distribution and, as the BDI-V and Monosyllabic Word Test scores at initial activation were not normally distributed, non-parametric correlations (Spearman rank) were conducted between BDI-V total scores at initial activation and the correct monosyllabic recognition percent score at the four timepoints. Median values are given as group averages. For comparisons of groups with different sample sizes, Kruskal–Wallis *H* statistics were used.

## Results

**Fig. 1:**
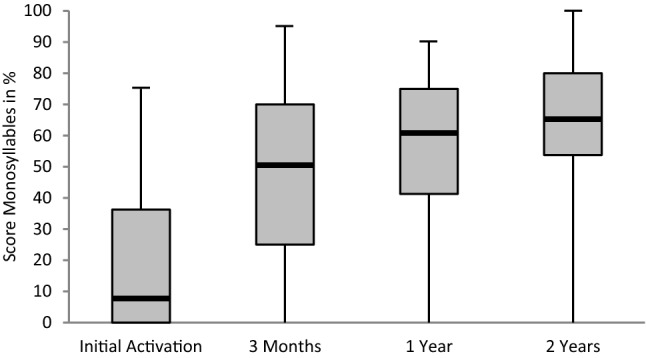
Boxplot representation for speech recognition score as measured with the Freiburg Monosyllabic Word Test at CI activation, 3 months, 1-year and 2-year follow-up visits

The development of speech recognition (FBE) over time is shown in Fig. 1. At the third day after initial activation, the patients reproduced on average 7.5% of the monosyllabic words. Three months later 50% correct words were reproduced, whereas 1 and 2 years later, the patients achieved 61.25 and 65% correct words, respectively. Of the 31 patients, one had missing audiological data at initial activation and one at 3 months after initial activation. Two patients missed the follow-up 1 year after initial activation but participated in the 2-year follow-up. One patient ceased rehabilitation voluntarily after the 3-month follow-up. In four patients, the 2-year follow-up had not taken place yet at the time of data analysis.

In some cases, patients did not reach any free speech comprehension (without cues) indicated by a value of 0 in the Monosyllabic Word Test. This was the case for about 50% of all patients at initial activation, which represents a normal course of CI rehabilitation.

In the BDI-V, the patients scored a median value of 13.00 (Min = 0, Max = 88). A total score of 18 corresponds to a percentile rank of 50.6 in the normative sample [24]. Nine patients reached a score of 35 and above, and as such, exhibited a high probability of a clinically relevant depression.

Statistically significant negative correlations were found between the BDI-V total score at initial activation and the correct monosyllable percent score 3 months after initial activation (*r* = − 0.456, *p* = 0.011) and 1 year after initial activation (*r* = − 0.472, *p* = 0.011; see Table 2). In contrast, the BDI-V total score and correct monosyllables both at initial activation were not correlated (*r* = − 0.227, *p* = 0.227). The correlation between the percent of correct monosyllables 2 years after initial activation and the BDI-V at initial activation did not reach significance (*r* = − 0.339, *p* = 0.090).

**Table 2:** Spearman correlations for the BDI-V score at initial activation of the CI and speech recognition measured at initial activation and 3 months, 1-year and 2-year follow-up visits

	BDI-V at Initial activation	*p*	*N*
Monosyll. after Init. Activation	− 0.227	0.227	30
Monosyll. after 3 Months	− **0.456**	**0.011**	30
Monosyll. after 1 Year	− **0.472**	**0.011**	28
Monosyll. after 2 Years	− 0.339	0.090	26

Significant correlations (*p* < 0.05) are highlighted in bold

In the present sample, no relations with age at initial activation were found for the BDI-V total score (*r* = − 0.120, *p* = 0.519), correct monosyllable percent score at initial activation (*r* = 0.164, *p* = 0.385) and 3 months after initial activation (*r* = 0.291, *p* = 0.119), and 1 year after initial activation (*r* = 0.321, *p* = 0.095). In contrast, the monosyllable percent score at 2 years after initial activation was correlated with age (*r* = 0.449, *p* = 0.022).

No significant differences were found between male (Md = 12.50) and female patients (Md = 13.00) in the BDI-V total score (Kruskal–Wallis *H*(1) = 0.866, *p* = 0.352).

The self-reported duration of severe to profound hearing loss was correlated with correct monosyllable percent score at 3 months (*r* = − 0.670, *p* < 0.001), 1 year (*r* = − 0.485, *p* = 0.022) and 2 years after initial activation (*r* = − 0.556, *p* = 0.011), but not related to the BDI total score (*r* = 0.133, *p* = 0.544).

To preclude that comorbid diseases had an influence on the relationship between BDI-V and speech recognition, differences between three patient groups regarding both variables were checked. Patients with no comorbid disease scored a median BDI-V value of 15.00, patients with chronic hypertension as the only comorbid disease scored a median value of 8.00, and patients with other comorbid diseases (see section Sample) scored a median value of 13.00. A Kruskal–Wallis test confirmed that there were no significant differences between groups in the BDI-V total score (*H*(2) = 1.201, *p* = 0.549). Likewise, these three groups did not differ in speech recognition at initial activation (*H*(2) = 0.097, *p* = 0.953), 3 months (*H*(2) = 0.104, *p* = 0.949), 1 year (*H*(2) = 0.557, *p* = 0.757) and 2 years (*H*(2) = 1.881, *p* = 0.390) after initial activation.

## Discussion

In the present study, the development of speech recognition after cochlear implantation was tracked up to 2 years, and related to the amount of depressive symptoms patients displayed at the time of initial activation of the implant. Results showed that the amount of depressive symptoms at initial activation was negatively correlated with the monosyllabic recognition score after 3 months and after 1 year of implant use.

Most previous studies concerned with the relationship between audiological outcome after cochlear implantation and psychological factors either tap psychological consequences of reduced or improved hearing [6–8, 10], or tap psychological predictors of audiological outcome other than depressive symptoms [9, 13, 14].

Brüggemann et al. [15] examined CI-patients with psychiatric diagnoses and found different hearing developments over time dependent on diagnosis.

To our knowledge, this is the first time that depressive symptoms in a wide range, i.e., also including non-psychiatric patients, could be related to later audiological outcome after cochlear implantation (the proportion of patients exceeding the cut-off for a probable clinically relevant depression is discussed below).

Possible explanations for this relationship comprise parameters that could influence audiological outcome indirectly, like motivation, compliance and habits of CI use, and on the other hand, there are conceivable parameters directly associated with speech recognition, like learning, memory and plasticity.

Depressive symptoms assessed with the BDI-V include apathy and fatigue [20]. In this respect, the impact of depressive symptoms on the development of speech recognition with the CI could be mediated by motivational factors and compliance. The individual outcome could be, among other factors, affected by the amount of speech comprehension training [e.g., 25, 26], as well as the constant use of the speech processor [27, 28], though this hypothesis remains to be validated with regard to the causal relationship [29]. Both training and use of the processor could be reduced with depressive symptoms, e.g., due to increased need of sleep and apathy. The amount of self-directed training, which is strongly recommended to patients during rehabilitation, was not controlled for in the present study. However, the daily use of the CI was controlled for by the processor-immanent data logging. Correlating the mean daily use in hours during the first 3 months with the BDI-V total score revealed a tendency, but not a significant relationship (*r* = − 0.389, *p* = 0.067), while the recognition of monosyllables after 3 months was negatively correlated with the mean daily use during the first 3 months (*r* = − 0.487, *p* = 0.022). Thus, the mean daily use could not be totally ruled out as an influencing factor, but cannot explain the present relationship between depressive symptoms and hearing outcome in large part.

Besides processor use and amount of training which could represent the quantity of learning possibilities, depressive symptoms could also influence the quality of learning the new way of hearing.

Studies with psychiatric populations have shown impairments in verbal memory and verbal learning in depression, which have been interpreted as problems in attention and in transfer to long-term memory [30]. Yet, impairments in verbal memory are verifiable only if patients displayed more than one depressive episode [31], and impaired verbal learning in depression is possibly dependent on anti-depressive medication [32]. Thus, a reduced acquirement of speech recognition after cochlear implantation caused by problems in verbal learning and memory is conceivable for patients with diagnosis of depression but not patients with moderate depressive symptoms.

However, impairments in verbal learning and memory have also been interpreted in the framework of the neuroplasticity hypothesis of depression [33], putting forward an altered long-term-depression and-potentiation in depression. This alteration has even been found in primary functions and regions using the example of visual evoked potentials [34]. Visual and auditory evoked potentials are supposed to be intensity-modulated by the serotonergic activity of the primary sensory cortices [35, 36]. In depression, the loudness dependence of the N1/P2 component in auditory evoked potentials can be used for prediction of reaction to antidepressant serotonin reuptake inhibitors [36]. Moreover, unmedicated depressed individuals without hearing loss do not only show a larger intensity-dependency, but also behavioral decrements in speech perception in noise [37]. Interestingly, in CI-patients, it was found that perception of monosyllabic words correlated positively with N1/P2 amplitudes [38]. Though yet speculative and limited by different experimental methods, the serotonergic activity in the primary auditory cortex might be a key underlying factor, linking the implications of depressive symptoms and speech processing via auditory perception. Again, the above mentioned results only apply to clinical diagnosis of (major) depression and not to moderate depressive symptoms, although chronic stress is a factor stated to have similar neural and neuropsychological effects [33, 34].

The present number of patients exceeding the cut off for a 90% probability of a clinically relevant depression corresponds to a percentage of 29%. The BDI-V alone does not provide a certain diagnosis and the cut off is a rough estimate which does not include this measurement error [24]. Nevertheless, the estimated number of depressive patients based on the BDI-V data would still be much higher than the global prevalence of depression [19], and even higher than the prevalence for a comorbid depression together with a chronic disease [39].

Li et al. [40] report a prevalence of moderate depression of about 14% for men and 20% for women with any self-reported hearing difficulties. Internal unpublished data from our center yield a proportion of about 18% patients above the BDI-V cut off at any timepoint during their treatment in the center.

The high percentage of potentially depressive persons in the present study could be due to the strict inclusion criteria involving only severe hearing loss in both sides, as Tambs [4] proposes a linear decline of mental health with increasing hearing loss. For the subjects who exceeded the suggested cut-off, the exact etiology of depressive symptoms remains unclear. Besides hearing loss, multiple factors could promote the emergence of depressive symptoms. Here, comorbid diseases could be excluded as such a possible factor.

However, to rule out that the results of the present study are confounded by two qualitatively different groups of patients, the analyses were repeated including only patients below the cut off (*N* = 22). In this subgroup, the BDI-V score and correct monosyllabic recognition in percent at 3 months and 2 years were normally distributed and yielded Pearson correlations of − 0.503 (*p* = 0.020) and − 0.446 (*p* = 0.049), respectively. This suggests that even in non-depressive patients, the amount of subclinical depressive symptoms is negatively associated with the development of speech recognition, and that this association could persist for a long period of time. Though the BDI originally was designed as an instrument for diagnosis of depression, the BDI-V is valid also in the subclinical range: Schmitt and Maes [20] investigated a representative sample without psychiatric history and found links between the BDI-V and job satisfaction as well as the belief in an unjust world.

Unfortunately, the depressive symptoms were not further tracked at the same timepoints as the audiological post measurements for all patients. Likewise, patients were not controlled for variables like therapeutic and or pharmacological antidepressant treatment at any time.

One could argue that the relationship between depressiveness and speech recognition could be due to underlying factors influencing both. Possible variables would be age [16, 24] and the duration of severe to profound hearing loss [2, 16]. Here, it could be shown that depressiveness was independent from both, while particularly duration of severe to profound hearing loss had an impact on speech recognition, as expected. Interestingly, in contrast to other studies, in the present sample, age at implantation was not a limiting factor to the development of speech recognition with CI. Moreover, age and sex did not have an impact on the BDI-V in the present sample. This finding is in contrast to general prevalence data [19] that shows a higher prevalence in women and an increase in prevalence until the ages around 60. The normative data of the BDI-V [24] displays higher values in women and an increase in the middle ages and a peak in very high ages. Though unexpected, due to the absence of these effects in the present sample it can be excluded that the relationship between depressiveness and speech recognition is valid only in subgroups of patients.

In future studies it should be resolved if a persisting impaired development of speech recognition with CI could be dependent on depressive symptoms only at initial activation or if it is due to stable depressive symptoms over time. Likewise, the effectiveness of interventions aimed at the depression regarding its impact on hearing development after cochlear implantation should be examined in further studies.

The present study has important clinical implications. To postpone implantation because of a diagnosed depression would not be an appropriate approach as a matter of course, for ethical reasons and because of the potentially positive effect of implantation on depression [6, 7, 10].

Care staff and CI-users should be sensitized to the link between depressive symptoms and the development of speech recognition with CI. CI recipients being at risk for depression at the time of implantation should be counselled regarding a suitable intervention.

## References

[1] Mathers CD, Smith A, Concha M (2003). Global burden of hearing loss in the year 2000.

[2] Arlinger S (2003). Negative consequences of uncorrected hearing loss-a review. Int J Audiol.

[3] Nachtegaal J, Smit JH, Smits C, Bezemer PD, van Beek JHM, Festen JM, Kramer SE (2009). The association between hearing status and psychosocial health before the age of 70 years: Results from an internet-based national survey on hearing. Ear Hear.

[4] Tambs K (2004). Moderate effects of hearing loss on mental health and subjective well-being: results from the Nord-Trondelag hearing loss study. Psychosom Med.

[5] Krueger B, Joseph G, Rost U, Strauß-Schier A, Lenarz T, Buechner A (2008). Performance groups in adult cochlear implant users: speech perception results from 1984 until today. Otol Neurotol.

[6] Knutson JF, Murray KT, Husarek S, Westerhouse K, Woodworth G, Gantz BJ, Tyler RS (1998). Psychological change over 54 months of cochlear implant use. Ear Hear.

[7] McRackan TR, Bauschard M, Hatch JL, Franko-Tobin E, Droghini HR, Velozo CA, Nguyen SA, Dubno JR (2018). Meta-analysis of cochlear implantation outcomes evaluated with general health-related patient-reported outcome measures. Otol Neurotol.

[8] Mo B, Lindbaek M, Harris S (2005). Cochlear implants and quality of life: a prospective study. Ear Hear.

[9] Mosnier I, Bebear JP, Marx M (2015). Improvement of cognitive function after cochlear implantation in elderly patients. JAMA Otolaryngol Head Neck Surg.

[10] Olze H, Szczepek AJ, Haupt H, Förster U, Zirke N, Gräbel S, Mazurek B (2011). Cochlear implantation has a positive influence on quality of life, tinnitus, and psychological comorbidity. Laryngoscope.

[11] Haas LJ (1990). Psychological safety of a multiple channel cochlear implant device: Psychological aspects of a clinical trial. Int J Technol Assess Health Care.

[12] Volleth N, Hast A, Lehmann EK, Hoppe U (2018). Subjective improvement of hearing through cochlear implantation [in German]. HNO.

[13] Knutson JF, Gantz BJ, Hinrichs JV, Schartz HA, Tyler RS, Woodworth G (1991) Psychological predictors of audiological outcomes of multichannel cochlear implants: preliminary findings. Ann Otol Rhinol Laryngol l00:817–822. 10.1177/000348949110001006 1952648

[14] Van Dijk JE, van Olphen AF, Langereis MC, Mens LH, Brokx JP, Smoorenburg GF (1999). Predictors of cochlear implant performance. Audiology.

[15] Brüggemann P, Szczepek AJ, Klee K, Gräbel S, Mazurek B, Olze H (2017). In patients undergoing cochlear implantation, psychological burden affects tinnitus and the overall outcome of auditory rehabilitation. Front Hum Neurosci.

[16] Blamey P, Artieres F, Başkent D (2013). Factors affecting auditory performance of postlinguistically deaf adults using cochlear implants: An update with 2251 patients. Audiol Neurotol.

[17] Hoppe U, Hast A, Hocke T, Iro H (2019). Maximum preimplantation monosyllabic score as predictor of cochlear implant outcome. HNO.

[18] Pisoni DB, Kronenberger WG, Harris MS, Moberly AC (2017). Three challenges for future research on cochlear implants. World J Otorhinolaryngol Head Neck Surg.

[19] World Health Organization (2017). Depression and other common mental disorders: Global health estimates. Geneva: World Health Organization. Licence: CC BY-NC-SA 3.0 IGO.

[20] Schmitt M, Maes J (2000). Vorschlag zur Vereinfachung des Beck-Depressions-Inventars (BDI). Diagnostica.

[21] Deutsches Institut für Normung (1973). DIN 45621:1973–10 Wörter für Gehörprüfung mit Sprache (DIN45621:1973).

[22] Beck AT, Steer RA (1987). Beck Depression Inventory (BDI).

[23] Schmitt M, Beckmann M, Dusi D, Maes J, Schiller A, Schonauer K (2003). Messgüte des vereinfachten Beck-Depressions-Inventars (BDI-V). Diagnostica.

[24] Schmitt M, Altstötter-Gleich C, Hinz A, Maes J, Brähler E (2006). Normwerte für das Vereinfachte Beck-Depressions-Inventar (BDI-V) in der Allgemeinbevölkerung. Diagnostica.

[25] Fu QJ, Galvin JJ (2008). Maximizing cochlear implant patients’ performance with advanced speech training procedures. Hear Res.

[26] Schumann A, Serman M, Gefeller O, Hoppe U (2015). Computer-based auditory phoneme discrimination training improves speech recognition in noise in experienced adult cochlear implant listeners. Int J Audiol.

[27] Francis HW, Chee N, Yeagle J, Cheng A, Niparko JK (2002). Impact of cochlear implants on the functional health status of older adults. Laryngoscope.

[28] Schvartz-Leyzac KC, Conrad CA, Zwolan TA (2019). Datalogging statistics and speech recognition during the first year of use in adult cochlear implant recipients. Otol Neurotol.

[29] Busch T, Vanpoucke F, Van Wieringen A (2017). Auditory environment across the life span of cochlear implant users: insights from data logging. J Speech Lang Hear Res.

[30] Marazziti D, Consoli G, Picchetti M, Carlini M, Faravelli L (2010). Cognitive impairment in major depression. Eur J Pharmacol.

[31] Fossati P, Harvey PO, Le Bastard G, Ergis AM, Jouvent R, Allilaire JF (2004). Verbal memory performance of patients with a first depressive episode and patients with unipolar and bipolar recurrent depression. J Psychiatr Res.

[32] Porter R, Gallagher P, Thompson J, Young A (2003). Neurocognitive impairment in drug-free patients with major depressive disorder. Br J Psychiatry.

[33] Nissen C, Holz J, Blechert J, Feige B, Riemann D, Voderholzer U, Normann C (2010). Learning as a model for neural plasticity in major depression. Biol Psychiatry.

[34] Normann C, Schmitz D, Fürmaier A, Döing C, Bach M (2007). Long-Term plasticity of visually evoked potentials in humans is altered in major depression. Biol Psychiatry.

[35] Hegerl U, Juckel G (1993). Intensity dependence of auditory evoked potentials as an indicator of central serotonergic neurotransmission: a new hypothesis. Biol Psychiatry.

[36] Gallinat J, Bottlender R, Juckel G, Munke-Puchner A, Stotz G, Kuss HJ, Mavrogiorgou P, Hegerl U (2000). The loudness dependency of the auditory evoked N1/P2-component as a predictor of the acute SSRI response in depression. Psychopharmacology.

[37] Gopal KV, Bishop CE, Carney L (2004). Auditory measures in clinically depressed individuals. II. Auditory evoked potentials and behavioral speech tests. Int J Audiol.

[38] Liebscher T, Alberter K, Hoppe U (2018). Cortical auditory evoked potentials in cochlear implant listeners via single electrode stimulation in relation to speech perception. Int J Audiol.

[39] Moussavi S, Chatterji S, Verdes E, Tandon A, Patel V, Ustun B (2007). Depression, chronic diseases, and decrements in health: results from the world health surveys. Lancet.

[40] Li C, Zhang X, Hoffman HJ, Cotch MF, Themann CL, Wilson MR (2014). Hearing impairment associated with depression in US adults, national health and nutrition examination survey 2005–2010. JAMA Otolaryngol Head Neck Surg.

